# Investigating the association between the tissue expression of miRNA‐101, JAK2/STAT3 with TNF‐α, IL‐6, IL‐1β, and IL‐10 cytokines in the ulcerative colitis patients

**DOI:** 10.1002/iid3.1224

**Published:** 2024-03-22

**Authors:** Qazaleh Voshagh, Amir Anoshiravani, Amin Karimpour, Golnaz Goodarzi, Sadra Samavarchi Tehrani, Ozra Tabatabaei‐Malazy, Ghodratollah Panahi

**Affiliations:** ^1^ Department of Clinical Biochemistry, School of Medicine Tehran University of Medical Sciences Tehran Iran; ^2^ Digestive Disease Research Center, Digestive Disease Research Institute Tehran University of Medical Sciences Tehran Iran; ^3^ Department of Pathobiology and Laboratory Sciences, School of Medicine North Khorasan University of Medical Sciences Bojnurd Iran; ^4^ Endocrine Research Center, Institute of Endocrinology and Metabolism Iran University of Medical Science Tehran Iran; ^5^ Non‐Communicable Diseases Research Center, Endocrinology and Metabolism Population Sciences Institute Tehran University of Medical Sciences Tehran Iran; ^6^ Endocrinology and Metabolism Research Center, Endocrinology and Metabolism Clinical Sciences Institute Tehran University of Medical Sciences Tehran Iran

**Keywords:** IBD, inflammation, JAK2‐STAT3, miRNA‐101, ulcerative colitis

## Abstract

**Background:**

Ulcerative colitis (UC) is a chronic inflammatory bowel disease caused by numerous factors, such as immune system dysfunction and genetic factors. MicroRNAs (miRNAs) play a crucial role in UC pathogenesis, particularly via the JAK‐STAT pathway. Our aim was to investigate the association between miRNA‐101 and JAK2‐STAT3 signaling pathway with inflammatory cytokines in UC patients.

**Methods:**

We enrolled 35 UC patients and 35 healthy individuals as the control group, referred to Shariati Hospital, Tehran, Iran. Patients were diagnosed based on clinical, laboratory, histological, and colonoscopy criteria. RNA and protein extracted from tissue samples. Real‐time PCR was used to assess the expression levels of miRNA‐101, interleukin (IL)‐1β, IL‐6, tumor necrosis factor (TNF)‐α, and IL‐10 genes, while western blot was employed to measure levels of P‐STAT3, total STAT3, and JAK2 proteins.

**Results:**

Expression of pro‐inflammatory cytokines TNF‐α, IL‐1β, and IL‐6 significantly increased, while the expression of IL‐10 significantly decreased in the case group versus controls. Additionally, miRNA‐101 expression was significantly higher in UC patients. A significant correlation between miRNA‐101 and IL‐6 expression was observed, indicating their relationship and possible impact on cell signaling pathways, JAK2‐STAT3. No significant changes were observed in phosphorylated and total STAT3 and JAK2 protein expression.

**Conclusion:**

This study provides evidence of increased miRNA‐101 expression in UC tissue, suggesting a potential correlation between miRNA‐101 and IL‐6 expression and their involvement in the JAK2‐STAT3 pathway. The study confirms alterations in UC patients' pro‐inflammatory cytokines and anti‐inflammatory IL‐10. However, further investigations are needed to understand the exact role of miRNA‐101 in UC pathogenesis fully.

## Graphical abstract

In western blot analysis, protein values were obtained through the ratio of target density/loading control (fold of control). The protein expression of the two groups was compared by using an unpaired *t*‐test. The values of the studied proteins in both patient and control groups did not change significantly, the *p*‐values of P‐STAT3, total STAT3, and JAK2 proteins were (*p* = .16), (*p* = .51), and (*p* = .4), respectively. The relative protein levels were normalized to the protein levels of β‐actin.

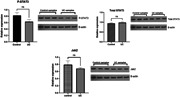

## 1 INTRODUCTION

Ulcerative colitis (UC) is a chronic inflammatory bowel disease (IBD) that is caused by aberrant immune system reactions that inflame the colon and the rectum. UC manifests as persistent inflammation within the gut's mucosal or submucosal layers. Its severity can vary, presenting symptoms such as stomach discomfort, frequent diarrhea, tiredness, loss of weight, bleeding from the rectum, and stools containing blood. In more extreme situations, individuals may notice blood and mucus in their stools, development of fistulas, fissures, and hemorrhoids, and suffer from anemia.^1^ This condition has been on a steady rise in industrialized nations, whereas in developing countries, there has been a sharp increase in incidence over recent decades.^2^ The highest incidence and prevalence rates have been recorded in Europe and America. Factors such as environmental and lifestyle influences, including the use of medications, diets high in sugar and fat, pollution, and stress, are linked to the emergence of the disease.^3^ The origin of IBD is not fully understood, but it is believed to stem from a complex interaction involving genetic predisposition, changes in the microbiome, and environmental influences. These factors contribute to a dysfunctional immune response and impair the integrity of the mucosal barrier.^4^

The immune response is crucial in UC's onset, escalation, and maintenance. Various cytokines and inflammatory mediators, known to play pivotal roles in UC, are linked to cellular responses, specifically classical cytokines such as tumor necrosis factor (TNF)‐α, interleukin (IL)‐1, and IL‐6. Cells implicated in the pathogenesis of UC include antigen‐presenting cells and T cells.^5^ In IBD, many cytokines drive chronic inflammation by activating the JAK–STAT signaling pathways.^6^ JAK2‐STAT3 is one of the most important pathways involved in the disease process.^7^ Based on studies, it has been proven that STAT3 is a critical factor in the disease and increases the process and pathogenesis of the disease.^8^

MicroRNAs (miRNAs) represent a category of noncoding RNAs distinguished by their roles in regulating gene expression at both transcriptional and posttranscriptional stages. The mature form of miRNAs consists of single‐stranded RNA molecules, typically 19 to 22 nucleotides in length.^9^ Research has shown that miRNAs regulate numerous cellular functions, including cell proliferation, programmed cell death (apoptosis), development, and metabolic processes.^9^, ^10^, ^11^ Moreover, miRNAs have been documented to influence innate and adaptive immune responses and inflammatory pathways across different cell types associated with UC.^12^ The miRNA‐101 is known as a tumor suppressor, and according to studies, it is involved in multiple biological processes related to cancer, such as proliferation, apoptosis, angiogenesis, and metastasis.^13^, ^14^, ^15^ It has been found that miRNA‐101 regulates the apoptosis of breast cancer cells and brain cell apoptosis through the JAK2‐STAT3 pathway by reducing JAK2 protein expression and inhibiting STAT3 protein.^16^, ^17^ According to studies, the IL‐6/STAT3 pathway is a critical factor in the development and progression of IBD to colorectal cancer (CAC). Several types of miRNAs, including miRNA‐101 play an essential role in inhibiting STAT3.^18^ In addition, it was found that miRNA‐101 is involved in the polarity of macrophages and increases the M1 inflammatory macrophages on the effect of pro‐inflammatory cytokines and causes more inflammatory responses.^19^

Considering the crucial function of miRNA‐101 in modulating the immune system, changes in the expression levels of miR‐101 could be linked to various human diseases.^20^ Considering the effective and regulatory role of miRNAs and the role of the JAK‐STAT pathway in the pathogenesis of UC, few studies have been conducted on the expression level of miRNA‐101 in IBD disease, and the role of this type of miRNA in UC disease has not been determined. In this study, our objective was to examine the expression level of miRNA‐101 in tissue samples from UC patients compared to healthy individuals and to explore how miRNA‐101 is associated with the activity of the JAK2‐STAT3 signaling pathway and the cytokines generated in patients with UC.

## 2 MATERIALS AND METHODS

### 2.1 Subjects

This study enrolled a total of 70 participants, comprising 35 individuals with UC and 35 healthy subjects, all aged between 42 and 64 years. Recruitment occurred at the gastroenterology and liver clinic of Tehran's Shariati Hospital, associated with Tehran University of Medical Sciences, during the period of 2021–2022. The inclusion criteria specified participants should be aged 18–65 years and not have a current or past diagnosis of infectious enterocolitis, colorectal cancer (CAC), diabetes, or, in the case of female participants, be pregnant or diagnosed with polycystic ovary syndrome. UC was diagnosed based on clinical, endoscopic, radiological, and histological criteria.

Personal information such as age, gender, medical history including glucose and lipid levels, medication use, and the affected area of UC was meticulously documented for each participant. Body mass index (BMI) was calculated using the formula of weight in kilograms divided by the square of height in meters. Clinical assessments, including fasting blood sugar (FBS), triglycerides (TG), low‐density lipoprotein cholesterol (LDL‐C), high‐density lipoprotein cholesterol (HDL‐C), and total cholesterol, were conducted to analyze the clinical profile of the participants. The comparison of clinical and biochemical parameters like BMI, FBS, and lipid profiles revealed no significant differences between the UC and control groups. Tissue samples were collected under professional supervision and were immediately stored at −80°C for preservation.

### 2.2 RNA extraction and cDNA synthesis

The RNA from tissue samples was isolated utilizing the Ana Cell RNA Extraction Kit, adhering strictly to the guidelines provided by the manufacturer. The synthesis of miRNA cDNA was accomplished in two stages with the aid of the Ana micro‐RNA Detection Kit, again following the manufacturer's detailed instructions. Initially, polyadenylation was carried out using an RT stem‐loop technique, which involved heating the samples at 70°C for 5 min. Following this, the reverse transcription process was executed using Moloney Murine Leukemia Virus Reverse Transcriptase, in line with the kit's specified protocols. For synthesizing cDNA from genes regulating cytokines, the Ana Cell cDNA Synthesis Kit was employed, according to the manufacturer's directives.

### 2.3 Quantitative real‐time PCR

Quantitative real‐time PCR was performed using Real Q Plus 2x Master Mix Green (Ampliqon). miR‐101 and miR‐u6 as an internal control forward primer were 5‐CCAGCGGTTACAGTACTGTGATA‐3 and 5‐AACGCTTCACGAATTTGCGT‐3, respectively. For inflammatory cytokines TNF‐α, IL‐1β, IL‐6 forward (F) and reverse (R) primers were F: 5‐CCAGGGACCTCTCTCTAATCA‐3 R:5‐TCAGCTTGAGGGTTTGCTAC‐3, F: 5‐GTACCTGTCCTGCGTGTTGA‐3 R:5‐GGGAACTGGGCAGACTCAAA‐3, F:5‐CCTGAACCTTCCAAAGATGGC‐3 R:5‐TTCACCAGGCAAGTCTCCTCA‐3, respectively. For IL‐10, forward and reverse primers were F:5‐TCAGGGTGGCGACTCTAT‐3 R:5‐TGGGCTTCTTTCTAAATCGTTC‐3, and β‐actin was used as an internal control in real‐time PCR and primers were F:5‐GAGCTACGAGCTGCCTGACG‐3 R:5‐GTAGTTTCGTGGATGCCACAG‐3. Quantitative Real‐Time PCR assays were conducted using a Rotor‐Gene Q instrument (Qiagen), utilizing 20 μL of PCR master mix for each reaction. The comparative CT method was employed to determine the relative levels of miR‐101 expression. To reduce experimental variability, all assays were performed in duplicate.

**Table:** 

Gene	Primer sequence	Product size (bp)
miR‐101	F: CCAGCGGTTACAGTACTGTGATA	
miR‐u6	F: AACGCTTCACGAATTTGCGT	
TNF‐α	F: CCAGGGACCTCTCTCTAATCA R: TCAGCTTGAGGGTTTGCTAC	106
IL‐1β	F: GTACCTGTCCTGCGTGTTGA R: GGGAACTGGGCAGACTCAAA	153
IL‐6	F: CCTGAACCTTCCAAAGATGGC R: TTCACCAGGCAAGTCTCCTCA	75
IL‐10	F: TCAGGGTGGCGACTCTAT R: TGGGCTTCTTTCTAAATCGTTC	199
β‐actin	F: GAGCTACGAGCTGCCTGACG R: GTAGTTTCGTGGATGCCACAG	120

### 2.4 Western blot

Colorectal mucosal tissues were homogenized in RIPA lysis buffer enriched with protease and phosphatase inhibitors (sourced from Beyotime) for protein extraction. The concentration of total proteins was quantified using the BCA protein assay kit from Thermo Scientific. Samples containing 50 μg of proteins each were subjected to SDS‐PAGE for separation and subsequently transferred to PVDF membranes (Millipore). These membranes were then blocked using 5% bovine serum albumin in TBST buffer at room temperature for 1 h before being incubated with specific primary antibodies overnight at 4°C. Following three washes with TBST, the membranes were exposed to HRP‐linked secondary antibodies for 1 h at room temperature. To enhance the visibility of the immunoreactive bands, ECL chemiluminescence detection reagents were applied, with the resulting signals captured and imaged using a detection system by BioRad. The antibodies used in this study were listed as follows: JAK2 (C‐10): sc‐390539, P‐STAT3: AB_10897947(BioLegend Cat. No. 651001), STAT3: AB_2565913 (BioLegend Cat. No. 678002), and β‐Actin (C4): sc‐47778. Protein values were obtained through the ratio of target density/loading control (fold of control).

### 2.5 Statistical analyses

Statistical evaluations were conducted with Prism software, version 9.5.1, presenting data as mean ± SD with significance set at *p* < .05. Variables not adhering to a normal distribution underwent log transformation before their analysis. Group comparisons utilized the unpaired Student's *t*‐test. The study also employed Pearson's correlation to assess the relationship between log‐transformed miRNA‐101 and various clinical and biochemical indicators, as well as its association with log‐transformed inflammatory cytokines. Furthermore, logistic regression analysis involving miRNA‐101 alongside age, BMI, TG, and total cholesterol was undertaken to ascertain if miRNA‐101 could serve as an indicator for predicting UC in the study population.

## 3 RESULTS

### 3.1 Demographic and biochemical characteristics of the subjects

This study included 35 individuals diagnosed with UC and 35 healthy participants. The participants' ages ranged from 42 to 64 years, with a notable difference in age distribution between the UC patients and the healthy control group, evidenced by a *p*‐value of .032. However, the study found no significant disparities in BMI with a *p*‐value of .6640, FBS levels (*p* = .567), total cholesterol levels (*p* = .133), TG (*p* = .369), HDL‐C (*p* = .875), and LDL‐C (*p* = .731) between the two groups, as detailed in Table 1.

**Table 1:** Clinical and biochemical characteristics of the study population.

Parameter	Control samples (*n* = 35)	UC samples (*n* = 35)	*p* Value
Age (year)	47.64 ± 5.8	56.8±8.1	.032
Gender (M/F)	16/19	18/17	‐
BMI (kg/m^2^)	23.31 ± 7.04	24.12 ± 7.06	.6640
FBS (mg/dL)	90.39 ± 11.02	88.37 ± 10.25	.5671
Total cholesterol (mg/dL)	159.2 ± 17.86	171.5 ± 34.96	.1334
TG (mg/dL)	123.7 ± 45.87	113.1 ± 49.05	.3699
HDL‐C (mg/dL)	45.08 ± 5.42	44.99 ± 6.4	.8759
LDL‐C (mg/dL)	99.86 ± 29.25	103 ± 21.87	.7316
Mesalazine (*n*)	‐	20	‐
Azathioprine (*n*)	‐	11	‐
Rhofanib (Tofacitinib) (*n*)	‐	4	‐
Conflict area (*n*)	Total = 20	Rectum = 7	Sigmoid = 8

Abbreviations: BMI, body mass index; FBS, fasting blood sugar; HDL‐C, high‐density lipoprotein cholesterol; LDL‐C, low‐density lipoprotein cholesterol; TG, triglycerides; UC, ulcerative colitis.

### 3.2 Tissue gene expression of TNF‐α, IL‐1β, IL‐6, and IL‐10 in UC and control subjects

**Figure 1:**
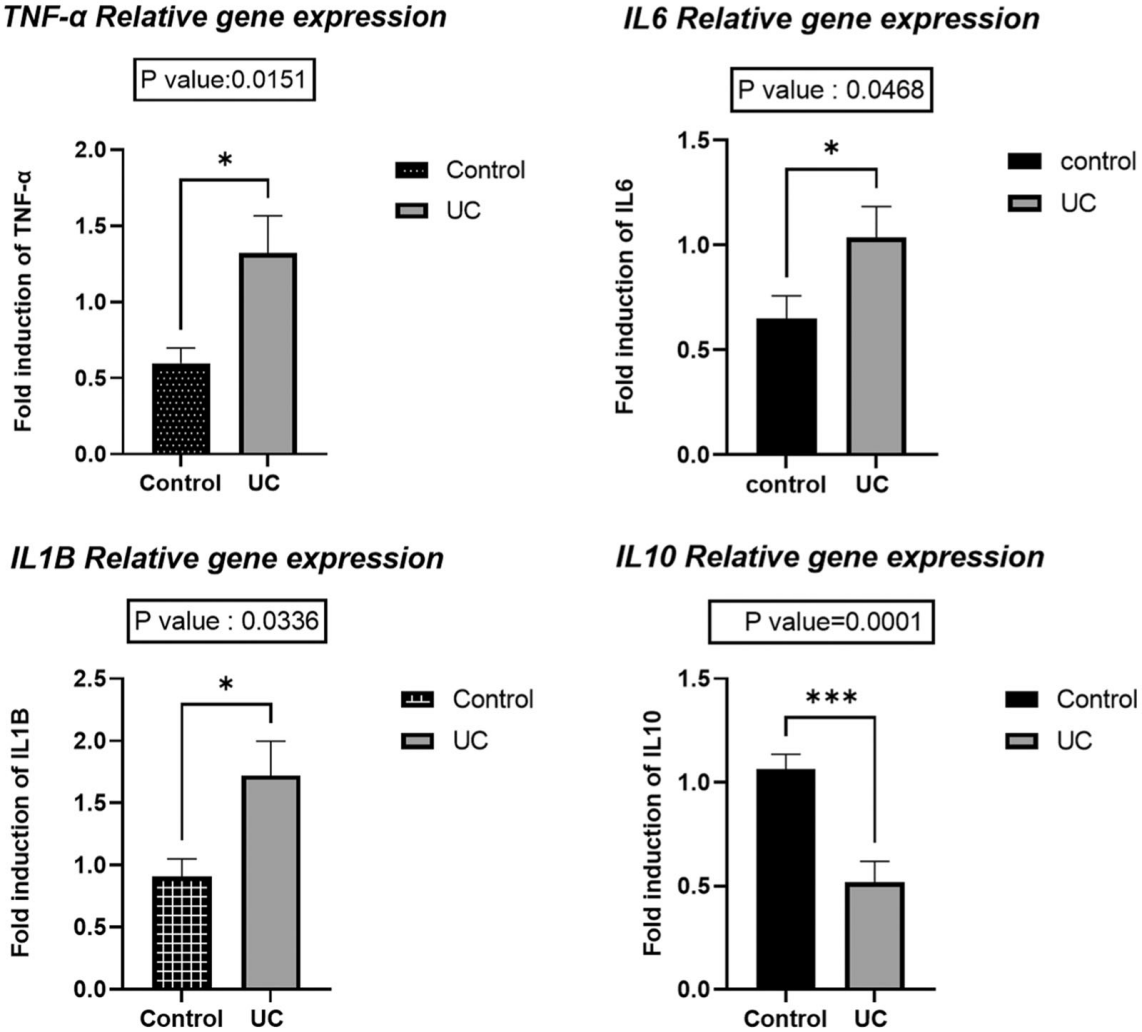
Level of tumor necrosis factor (TNF)‐α, interleukin (IL)‐1β, IL‐6, and IL‐10 in ulcerative colitis (UC) and control subjects.

The study found that the gene expression levels of TNF‐α, IL‐1β, and IL‐6 were significantly elevated in the tissue samples of UC patients compared to those of the control group. Specifically, the gene expression of TNF‐α was higher with a *p*‐value of .0151, IL‐6 was increased with a *p*‐value of .0468, and IL‐1β showed a higher level with a *p*‐value of .0336 in UC patients' tissues relative to healthy individuals. Conversely, the expression level of the IL‐10 gene was notably reduced in UC samples compared to control subjects, with a significant *p*‐value of .0001, indicating a decrease in anti‐inflammatory cytokine levels in the context of UC. The relative expression levels of these genes were normalized against the expression of β‐actin to ensure accuracy in measurement (Figure 1).

### 3.3 Tissue gene expression of miRNA‐101 in UC and control subjects

**Figure 2:**
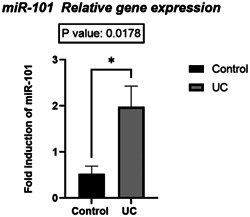
Level of microRNA (miRNA)‐101 in ulcerative colitis (UC) and control subjects.

The expression level of the miRNA‐101 gene was different in the two investigated groups and according to the statistical analysis, the expression level of this gene was increased in UC samples compared to control subjects (*p* = .0178). The relative expression levels were normalized to the expression of miR‐U6 (Figure 2).

### 3.4 Correlation of miRNA‐101 tissue expression with biochemical and clinical parameters

After observing changes in the expression of miRNA‐101 in tissue samples of UC patients compared to control subjects, it was decided to use Pearson's indicator to evaluate the correlation between the logarithm (∆Ct) of miRNA‐101 with different clinical and biochemical parameters. There was no significant relationship between the expression of miRNA‐101 logarithm between clinical and biochemical factors in UC and control subjects.

### 3.5 Correlation of miRNA‐101 gene with TNF‐α, IL‐6, IL‐1β, and IL‐10 genes

The study found a significant correlation between the log‐transformed expression levels of miRNA‐101 and the IL‐6 gene in both UC patients (*p* = .0001) and control subjects (*p* = .012). However, no significant correlation was observed between them.

### 3.6 Protein expression of P‐STAT3, STAT3, and JAK2

**Figure 3:**
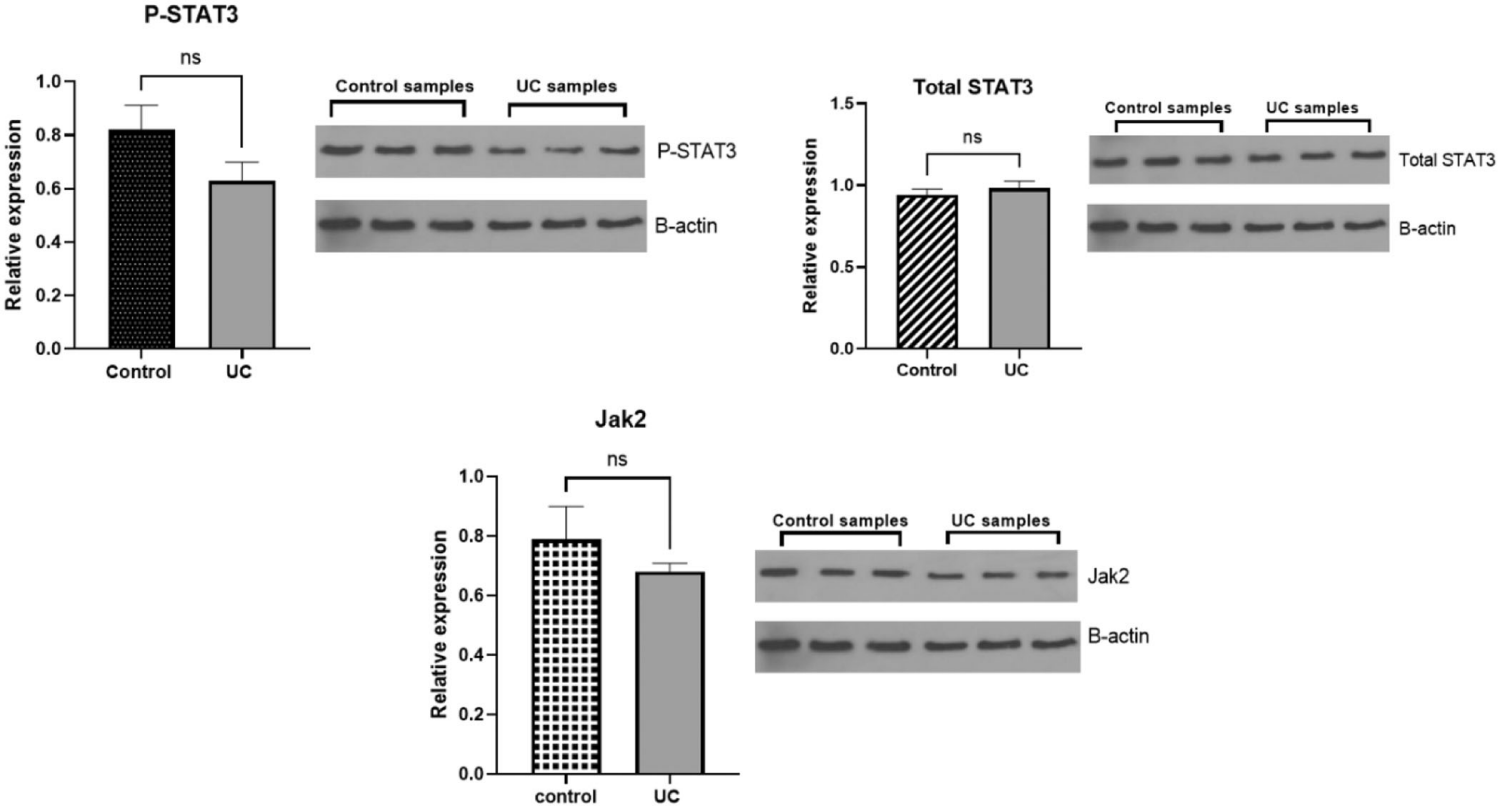
Protein level of P‐STAT3, STAT3, and JAK2 in ulcerative colitis (UC) and control subjects. The two groups' protein expression was compared using appropriate statistical tests (unpaired *t*‐test).

The values of the studied proteins in both patient and control groups did not change significantly (Figure 3); the *p*‐values of P‐STAT3, total STAT3, and JAK2 proteins were (*p* = .16, .51, and .4), respectively. The relative protein levels were normalized to the protein levels of β‐actin (Figure 3).

### 3.7 Logistic regression analysis of miRNA‐101

Among the five factors included (age, BMI, TG, total cholesterol, and miRNA‐101), none had a significant relationship with UC. Although the age factor had an OR less than 1 (0.68), which reduction increases the risk of UC, the obtained *p*‐value (*p* = .07) was not significant.

## 4 DISCUSSION

In recent years, considerable efforts have been made to determine the potential of miRNAs as biomarkers for diagnosis, prognosis, and therapeutic targets across various diseases. This study aimed to explore the relationship between miRNA‐101, the JAK2‐STAT3 signaling pathway, and inflammatory cytokines in the context of UC.

The current study revealed a significant elevation in the expression of pro‐inflammatory cytokines such as TNF‐α, IL‐1β, and IL‐6 among patients with UC in comparison to the control group. Given the critical function these cytokines play in IBD, particularly UC, exploring their gene expression is crucial. This observation aligns with previous reports indicating abnormal levels of TNF‐α, IL‐1β, and IL‐6 expression in UC patient tissues. Olsen et al.^21^ demonstrated that elevated levels of TNF‐α are associated with UC and play a role in multiple inflammatory mechanisms. Increased mRNA expression of TNF‐α is related to tissue inflammation in samples of CD and UC patients,^22^ and it was demonstrated that TNF‐α activates protein kinase and the cellular pathway (NF)‐κB, which ultimately leads to cell differentiation, proliferation, and increases the production of pro‐inflammatory cytokines. As a result, increased expression of TNF‐α causes mucosal barrier defects in UC patients.^23^ Interleukin‐1β produced by macrophages. It is involved in the pathogenesis of UC.^24^ Stevens et al.^25^ showed that increased mRNA expression of IL‐1β was seen in tissue UC samples compared to CD. Also, aberrant amounts of IL‐6 are found in serum samples and intestinal tissue biopsies in UC patients. In studies, an increase of this cytokine has been seen in the mucosal tissue of UC patients.^26^ Contrary to the results obtained in our study, in IBD patients, in addition to the increase of pro‐inflammatory cytokines, high levels of IL‐10 were also reported in UC compared to control samples.^26^, ^27^ In this present study, we demonstrated that the gene expression of IL‐10 decreased significantly in patients compared to the control group. In support of this finding, in similar studies, reduced expression of IL‐10 was reported in the tissue of UC patients compared to controls.^28^, ^29^ Also, Funakoshi et al.^30^ demonstrated that the decreased expression of IL‐10 in UC patients was significantly higher than CD.

Our study revealed a significant elevation in the expression of the miRNA‐101 gene in patients compared to the control group. Supporting this result, Schaefer et al.^31^ also observed heightened levels of miRNA‐101 in both tissue and serum of individuals with UC. Also, Nijakowski and Surdacka^32^ reported an increase in miRNA‐101 gene expression in IBD patients. Also, in this study, we demonstrated the correlation between miRNA‐101 and IL‐6, which is in line with Several recent studies. Gao et al.^33^ demonstrated miRNA‐101 as a pro‐inflammatory and proapoptotic miRNA that suppresses and inhibits the expression of MKP1 and increases TNF‐α, IL‐6, and IL‐1β levels in response to the effect of pro‐inflammatory lipopolysaccharides. Also, Zhang et al.^34^ showed that treatment with IL‐6 in a THP‐1‐derived macrophage cell line causes a significant increase in the expression of miRNA‐101 in this cell line and somehow indicates the relationship between IL‐6 and miRNA‐101.

According to the results obtained from this study, the phosphorylation of STAT3 was unchanged. Total protein expression of STAT3 and JAK2 was not significantly different in UC samples compared to control. In contrast, other studies reported that expression and phosphorylation of STAT3 and gene expression of STAT3 and JAK2 is increased in UC patients.^35^ Also, it was reported that increase protein levels of STAT3 in UC tissue samples related to the severity of disease.^36^ According to the studies, STAT3 is the main STAT in IBD, and the phosphorylation of STAT3 by JAK1,2 proteins through the signaling of gp130 family cytokines such as IL‐6 and IL‐10 plays an important role in UC pathogenesis.^37^ According to studies, the IL‐6/STAT3 pathway is a critical factor in the development and progression of IBD to CAC.^38^

On the other hand, STAT3 activated by IL‐10 has a protective role against IBD, inhibiting the progression of UC.^39^ Furthermore, according to studies, miRNA‐101 can indirectly affect the expression of STAT3 through its inhibitory effect on EZH2, which is a part of PRC2 that is involved in inhibiting gene expression. The changes of this protein have been seen in pediatric UC,^40^ while in Guo et al.^16^ study, it has been found that miRNA‐101 has a direct effect on the 3′‐UTR of JAK2 protein in brain injuries.

Overall, these findings suggest that no changes in the protein level of P‐STAT3, STAT3, and JAK2 in our study can be due to the effect of miRNA‐101 and the combined effect of pro‐inflammatory cytokines and the reduction of anti‐inflammatory cytokine (IL‐10) on these proteins. Taken together, increased expression of miRNA‐101 has inhibited STAT3 phosphorylation and decreased JAK2 expression; besides, increased expression of pro‐inflammatory cytokines, especially IL‐6, increases STAT3 phosphorylation and activates the JAK2‐STAT3 pathway. Also, due to the reduced expression of IL‐10, the effect of this cytokine was not seen on the JAK2‐STAT3 pathway. On the other hand, consuming medications used in UC patients can affect the expression of JAK2 proteins and the phosphorylation of STAT3.

The study faced several limitations, including a relatively small sample size, potentially affecting the broader applicability of its findings. Furthermore, it focused solely on the expression levels of miRNA‐101 and a few inflammatory cytokines, overlooking additional molecular mechanisms and pathways that might play a role in UC's etiology. To enhance the validity of these preliminary findings, future research should incorporate functional studies alongside larger and more varied participant groups. It is advisable for subsequent studies to explore the expression of miRNA‐101 across various stages of UC.

## 5 CONCLUSIONS

Our data demonstrate that the mRNA expression levels of pro‐inflammatory cytokines, including TNF‐α, IL‐1β, IL‐6, as well as miRNA‐101, are elevated. In contrast, the expression of IL‐10 is reduced in patients with UC compared to controls. These findings are consistent with previous research. Additionally, the absence of changes in the protein expression levels of phosphorylated STAT3, total STAT3, and JAK2 may be attributed to the combined effects of pro‐inflammatory and anti‐inflammatory cytokines and the influence of miRNA‐101 and medications administered to UC patients. The observed correlation between IL‐6 and miRNA‐101 in our study subjects suggests a significant association between these two analytes, warranting further investigation to elucidate the precise role of this miRNA in the pathogenesis of UC.

## AUTHOR CONTRIBUTIONS

**Qazaleh Voshagh**: Data curation; formal analysis, writing—original draft. **Amir Anoshiravani**: Formal analysis; writing—review & editing. **Amin Karimpour**: Formal analysis; writing—review & editing. **Golnaz Goodarzi**: Formal analysis; writing—review & editing. **Sadra Samavarchi Tehrani**: Formal analysis; writing—review & editing. **Ozra Tabatabaei‐Malazy**: Conceptualization; investigation; supervision; writing—review & editing. **Ghodratollah Panahi**: Conceptualization; funding acquisition; investigation; project administration; supervision; writing—review & editing. All authors read and approved the final manuscript.

## CONFLICT OF INTEREST STATEMENT

The authors declare no conflict of interest.

## ETHICS STATEMENT

The Ethics Review Board of the Tehran University of Medical Sciences approved the study, and informed consent was obtained from all participants. The study received approval from the Ethics Review Board of the Tehran University of Medical Sciences (approval number IR.TUMS.MEDICINE.REC.1401.725), and informed consent was secured from all subjects. It is confirmed that “individual authors” residing in the country under sanctions (Iran) are not on any numbered lists or from North Korea, Crimea or South Sudan.

## Data Availability

All data produced in the present study are available to the corresponding authors upon reasonable request.

## References

[1] Head KA , Jurenka JS . Inflammatory bowel disease part I: ulcerative colitis‐pathophysiology and conventional and alternative treatment options. Altern Med Rev. 2003;8(3):247‐283. 12946238

[2] Kaplan GG , Ng SC . Understanding and preventing the global increase of inflammatory bowel disease. Gastroenterology. 2017;152(2):313‐321. 27793607 10.1053/j.gastro.2016.10.020

[3] Shouval DS , Rufo PA . The role of environmental factors in the pathogenesis of inflammatory bowel diseases: a review. JAMA Pediatr. 2017;171(10):999‐1005. 28846760 10.1001/jamapediatrics.2017.2571

[4] De Souza HSP , Fiocchi C . Immunopathogenesis of IBD: current state of the art. Nat Rev Gastroenterol Hepatol. 2016;13(1):13‐27. 26627550 10.1038/nrgastro.2015.186

[5] Kmieć Z , Cyman M , Ślebioda TJ . Cells of the innate and adaptive immunity and their interactions in inflammatory bowel disease. Adv Med Sci. 2017;62(1):1‐16. 28126697 10.1016/j.advms.2016.09.001

[6] Schreiner P , Neurath MF , Ng SC , et al. Mechanism‐based treatment strategies for IBD: cytokines, cell adhesion molecules, JAK inhibitors, gut flora, and more. Inflamm Intest Dis. 2019;4(3):79‐96. 31559260 10.1159/000500721 PMC6751442

[7] Zundler S , Neurath M . Integrating immunologic signaling networks: the JAK/STAT pathway in colitis and colitis‐associated cancer. Vaccines. 2016;4(1):5. 26938566 10.3390/vaccines4010005 PMC4810057

[8] Wang S , Shen L , Luo H . Identification and validation of key miRNAs and a microRNA‐mRNA regulatory network associated with ulcerative colitis. DNA Cell Biol. 2021;40(2):147‐156. 33347387 10.1089/dna.2020.6151

[9] Kawaguchi T , Komatsu S , Ichikawa D , et al. Circulating microRNAs: a next‐generation clinical biomarker for digestive system cancers. Int J Mol Sci. 2016;17(9):1459. 27598137 10.3390/ijms17091459 PMC5037738

[10] Arias N , Aguirre L , Fernández‐Quintela A , et al. MicroRNAs involved in the browning process of adipocytes. J Physiol Biochem. 2016;72:509‐521. 26695012 10.1007/s13105-015-0459-z

[11] Prattichizzo F , Giuliani A , De Nigris V , et al. Extracellular microRNAs and endothelial hyperglycaemic memory: a therapeutic opportunity? Diabetes Obes Metab. 2016;18(9):855‐867. 27161301 10.1111/dom.12688 PMC5094499

[12] Toyonaga T , Saruta M . Role of microRNAs in the pathophysiology of ulcerative colitis. Immuno. 2021;1(4):558‐573.

[13] Liu X , Tang H , Chen J , et al. MicroRNA‐101 inhibits cell progression and increases paclitaxel sensitivity by suppressing MCL‐1 expression in human triple‐negative breast cancer. Oncotarget. 2015;6(24):20070‐20083. 26036638 10.18632/oncotarget.4039 PMC4652988

[14] Zhang J , Guo JF , Liu DL , Liu Q , Wang JJ . MicroRNA‐101 exerts tumor‐suppressive functions in non‐small cell lung cancer through directly targeting enhancer of zeste homolog 2. J Thorac Oncol. 2011;6(4):671‐678. 21270667 10.1097/JTO.0b013e318208eb35

[15] Zhang K , Zhang Y , Ren K , Zhao G , Yan K , Ma B . MicroRNA‐101 inhibits the metastasis of osteosarcoma cells by downregulation of EZH2 expression. Oncol Rep. 2014;32(5):2143‐2149. 25190211 10.3892/or.2014.3459

[16] Guo X , Shen X , Yong Z . MiR‐101 protects against the cerebral I/R injury through regulating JAK2/STAT3 signaling pathway. Neuropsychiatr Dis Treat. 2021;17:2791‐2802. 34465995 10.2147/NDT.S292471 PMC8403023

[17] Wang L , Li L , Guo R , et al. miR‐101 promotes breast cancer cell apoptosis by targeting janus kinase 2. Cell Physiol Biochem. 2014;34(2):413‐422. 25059472 10.1159/000363010

[18] Li Y , de Haar C , Chen M , et al. Disease‐related expression of the IL‐6/STAT3/SOCS3 signalling pathway in ulcerative colitis and ulcerative colitis‐related carcinogenesis. Gut. 2010;59(2):227‐235. 19926618 10.1136/gut.2009.184176

[19] Hübenthal M , Franke A , Lipinski S , Juzėnas S . MicroRNAs and inflammatory bowel disease. In: Molecular Genetics of Inflammatory Bowel Disease, 2019:203‐230.

[20] Wang CZ , Deng F , Li H , et al. MiR‐101: a potential therapeutic target of cancers. Am J Transl Res. 2018;10(11):3310‐3321. 30662588 PMC6291716

[21] Olsen T , Goll R , Cui G , et al. Tissue levels of tumor necrosis factor‐alpha correlates with grade of inflammation in untreated ulcerative colitis. Scand J Gastroenterol. 2007;42(11):1312‐1320. 17852866 10.1080/00365520701409035

[22] Gomes RG , Brito CAA , Martinelli VF , et al. HLA‐G is expressed in intestinal samples of ulcerative colitis and Crohn's disease patients and HLA‐G5 expression is differentially correlated with TNF and IL‐10 cytokine expression. Hum Immunol. 2018;79(6):477‐484. 29588183 10.1016/j.humimm.2018.03.006

[23] Pedersen J . Inflammatory pathways of importance for management of inflammatory bowel disease. World J Gastroenterol. 2014;20(1):64. 24415859 10.3748/wjg.v20.i1.64 PMC3886034

[24] Ashwood P , Harvey R , Verjee T , Wolstencroft R , Thompson RPH , Powell JJ . Titel. Inflamm Res. 2004;53:53‐59. 15021969 10.1007/s00011-003-1219-z

[25] Stevens C , Walz G , Singaram C , et al. Tumor necrosis factor‐α, interleukin‐1β, and interleukin‐6 expression in inflammatory bowel disease. Dig Dis Sci. 1992;37:818‐826. 1587185 10.1007/BF01300378

[26] Matsuda R , Koide T , Tokoro C , et al. Quantitive cytokine mRNA expression profiles in the colonic mucosa of patients with steroid naïve ulcerative colitis during active and quiescent disease. Inflamm Bowel Dis. 2009;15(3):328‐334. 18942752 10.1002/ibd.20759

[27] Autschbach F , Braunstein J , Helmke B , et al. In situ expression of interleukin‐10 in noninflamed human gut and in inflammatory bowel disease. Am J Pathol. 1998;153(1):121‐130. 9665472 10.1016/S0002-9440(10)65552-6 PMC1852930

[28] Nielsen OH , Køppen T , Rüdiger N , Horn T , Eriksen J , Kirman I . Involvement of interleukin‐4 and‐10 in inflammatory bowel disease. Dig Dis Sci. 1996;41:1786‐1793. 8794795 10.1007/BF02088746

[29] Schreiber S , Heinig T , Thiele HG , Raedler A . Immunoregulatory role of interleukin 10 in patients with inflammatory bowel disease. Gastroenterology. 1995;108(5):1434‐1444. 7729636 10.1016/0016-5085(95)90692-4

[30] Funakoshi K , Sugimura K , Anezaki K , Bannai H , Ishizuka K , Asakura H . Spectrum of cytokine gene expression in intestinal mucosal lesions of Crohn's disease and ulcerative colitis. Digestion. 1998;59(1):73‐78. 9468102 10.1159/000007470

[31] Schaefer JS , Attumi T , Opekun AR , et al. MicroRNA signatures differentiate Crohn's disease from ulcerative colitis. BMC Immunol. 2015;16:5. 25886994 10.1186/s12865-015-0069-0 PMC4335694

[32] Nijakowski K , Surdacka A . Salivary biomarkers for diagnosis of inflammatory bowel diseases: a systematic review. Int J Mol Sci. 2020;21(20):7477. 33050496 10.3390/ijms21207477 PMC7589027

[33] Gao Y , Liu F , Fang L , Cai R , Zong C , Qi Y . Genkwanin inhibits proinflammatory mediators mainly through the regulation of miR‐101/MKP‐1/MAPK pathway in LPS‐activated macrophages. PLoS One. 2014;9(5):e96741. 24800851 10.1371/journal.pone.0096741 PMC4011752

[34] Zhang N , Lei J , Lei H , et al. MicroRNA‐101 overexpression by IL‐6 and TNF‐α inhibits cholesterol efflux by suppressing ATP‐binding cassette transporter A1 expression. Exp Cell Res. 2015;336(1):33‐42. 26033364 10.1016/j.yexcr.2015.05.023

[35] Bai A , Hu P , Chen J , et al. Blockade of STAT3 by antisense oligonucleotide in TNBS‐induced murine colitis. Int J Colorectal Dis. 2007;22:625‐635. 17089128 10.1007/s00384-006-0229-z

[36] Li F , Zou Y , Li X . Up‐regulation of signal transducer and activator of transcription‐3 is associated with aggravation of ulcerative colitis. Surgeon. 2010;8(5):262‐266. 20709283 10.1016/j.surge.2010.03.003

[37] Schwartz DM , Kanno Y , Villarino A , Ward M , Gadina M , O'Shea JJ . JAK inhibition as a therapeutic strategy for immune and inflammatory diseases. Nat Rev Drug Discov. 2017;16(12):843‐862. 29104284 10.1038/nrd.2017.201

[38] Grivennikov S , Karin E , Terzic J , et al. IL‐6 and Stat3 are required for survival of intestinal epithelial cells and development of colitis‐associated cancer. Cancer Cell. 2009;15(2):103‐113. 19185845 10.1016/j.ccr.2009.01.001 PMC2667107

[39] Alonzi T . Induced somatic inactivation of STAT3 in mice triggers the development of a fulminant form of enterocolitis. Cytokine. 2004;26(2):45‐56. 15050604 10.1016/j.cyto.2003.12.002

[40] Koukos G , Polytarchou C , Kaplan JL , et al. MicroRNA‐124 regulates STAT3 expression and is down‐regulated in colon tissues of pediatric patients with ulcerative colitis. Gastroenterology. 2013;145(4):842‐852. 23856509 10.1053/j.gastro.2013.07.001 PMC4427058

